# Ultrasound-promoted preparation of polyvinyl ferrocene-based electrodes for selective formate separation: Experimental design and optimization

**DOI:** 10.1016/j.ultsonch.2022.106146

**Published:** 2022-08-30

**Authors:** Sevgi Polat, Ruud Kortlever, Huseyin Burak Eral

**Affiliations:** a Complex Fluid Processing Section, Process & Energy Department, Faculty of Mechanical, Maritime and Materials Engineering, Delft University of Technology, 2628 CB Delft, The Netherlands; b Chemical Engineering Department, Faculty of Engineering, Marmara University, 34854 İstanbul, Turkey; c Large-Scale Energy Storage Section, Process & Energy Department, Faculty of Mechanical, Maritime and Materials Engineering, Delft University of Technology, 2628 CB Delft, The Netherlands

**Keywords:** Electrochemical separations, Ultrasound, Redox electrode, Optimization, Experimental design

## Abstract

The selective separation of ions is a major technological challenge having far-ranging impacts from product separation in electrochemical production of base chemicals from CO_2_ to water purification. In recent years, ion-selective electrochemical systems leveraging redox-materials emerged as an attractive platform based on their reversibility and remarkable ion selectivity. In the present study, we present an ultrasound-intensified fabrication process for polyvinyl ferrocene (PVF)–functionalized electrodes in a carbon nanotube (CNT) matrix for selective electro-adsorption of formate ions. To this end, a response surface methodology involving the Box–Behnken design with three effective independent variables, namely, PVF to CNT ratio, sonication duration, and ultrasonic amplitude was applied to reach the maximum formate adsorption efficiency. The fabricated electrodes were characterized using cyclic voltammetry, X-ray diffraction, Raman spectroscopy, and scanning electron microscopy (SEM). SEM images revealed that an optimized ultrasonic amplitude and sonication time provided remarkable improvements in electrode morphology. Through a sedimentation study, we qualitatively demonstrate that the main optimized conditions improved PVF/CNT dispersion stability, consequently providing the highest number of active surface sites for adsorption and the highest adsorption efficiency. The highest percentage of active electrode surface sites and the maximum adsorption efficiency were 97.8 and 90.7% respectively at a PVF/CNT ratio of 3, ultrasonication time of one hour, and 50% ultrasonic amplitude.

## Highlights

• Optimization for ultrasound-assisted fabrication of PVF/CNT electrodes was performed.
• Ultrasonic treatment enhanced the stability of fabricated PVF/CNT electrodes.
• Ultrasound had a pronounced effect on the surface morphology of PVF/CNT electrodes.
• The Box–Behnken experimental design was applied to determine optimum active surface sites.
• The efficiency of formate adsorption was improved by optimizing experimental conditions.

## Graphical abstract

**Figure:**
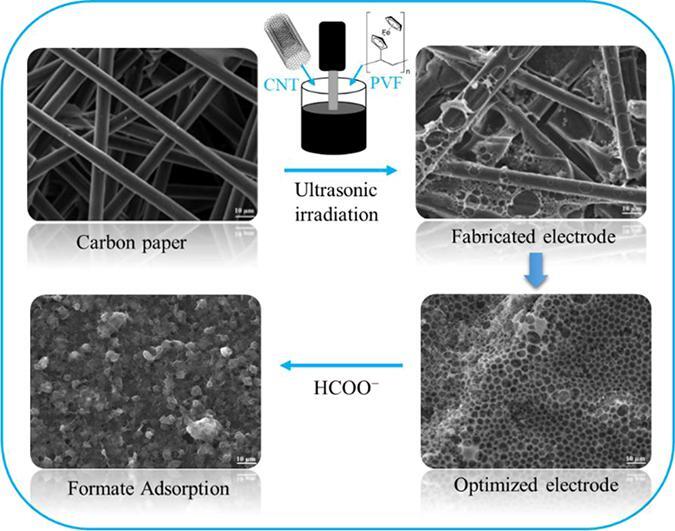

## 1 Introduction

Over the past decades, the electrochemical reduction of carbon dioxide (CO_2_) into industrially valuable products has become one of the most promising for valorizing anthropogenic CO_2_ emissions, while providing a means of energy storage for intermittent renewable sources, such as wind and solar [1], [2], [3], [4], [5]. One of the interesting target products for CO_2_ reduction is formate, as it has the potential to generate the highest revenue per mole of electrons consumed [6]. Although the electrochemical reduction of CO_2_ into valuable products is promising, product separation remains a challenge as most of the dissolved products are present at low concentrations (mostly in the millimolar to nanomolar concentration range) along with a significant excess of electrolyte anions [7], [8], [9].

Beyond the electrochemical reduction of CO_2_, the selective separation of organic anions offers opportunities in chemical and pharmaceutical industries [10]. In the chemical industry, major catalytic processes involve the use of metal catalysts or inorganic salts to produce a range of charged organic fragments. These catalytic processes and the subsequent separation steps are the most cost-intensive parts of the production process. In the pharmaceutical industry, production costs are increased when multistep organic syntheses are used to synthesize the desired compound due to the limiting yields of sequential reactions; therefore, even minor improvements to recovery can translate into major cost benefits. In both industries, as in the electrochemical CO_2_ reduction process, very low quantities of organic charged species are produced compared to that of their competing ions, such as electrolytes, buffers, side products, or catalysts; therefore, selectively separating these carboxylates, sulfonates, and phosphonates found in many micropollutants of concern in pharmaceuticals, and other products from catalytic chemical processes is a major challenge [11], [12], [13], [14]. Some organic ions, such as naturally occurring and synthetic carboxylates, can be of scientific and practical importance. One such ion, formate (HCOO^−^), the smallest carboxylate, is a model representative of this class of compounds. Because formate is a key product in the CO_2_ utilization pathway, its separation from homogeneous reaction mixtures is a major technological challenge [15], [16]. Selective separation of formate ions has the potential to not only contribute to the development of CO_2_ reduction technology but also in the remediation of pollutants to produce clean water. It has the potential to contribute to addressing sustainable development goals put forward by the United Nations [17]. In recent years, the selective separation of formate ions over competing ions using electrochemical separation techniques emerged as an popular option owing to the fast adsorption/desorption kinetics involved, fine control over solute molecules by an electrical potential, modularity, and its reusability [12], [18], [19].

Pseudo-capacitive deionization (PCDI), an electrochemical separation technique capable of selectively capturing ions, is based on heterogeneous redox-active organometallic compounds deposited on the surface of a carbon electrode. The efficiency of PCDI process is closely related to the ability to create desired structure–function relationships by tuning a chemical system at an atomistic level [20]. Mao et al. [21] have reported that integration of a metallocene polymer, polyvinyl ferrocene (PVF), with carbon nanotubes (CNTs) functions as a supercapacitor. Increased electrochemical performance and selectivity for a nonspecific surface are possible by the functionalization of porous electrodes. Su et al. [12] have applied this method to selectively capture carboxylates, sulfonates, and phosphonates from other inorganic anions, often used as electrolytes in aqueous solutions. This study [12] reports that formate is adsorbed selectively on the functionalized electrode by self-assembly of PVF and CNT dispersed in chloroform assisted by an ultrasonic bath. Although Su et al. [12] reports successful separation of formate from competing anions, the dispersion of CNTs remains a challenge for their utilization in electrochemical testing [22]. The dispersion quality of the PVF/CNT could seriously affect the performance of the fabricated electrode. The stability of the PVF/CNT suspension is a concern that must be addressed to further develop this technology. It has been shown that ultrasound waves enhance the dispersion quality and stability of the suspension due to their cavitation effect [23]. These waves promote electrochemical reactions resulting in improved efficiency, higher yields, and faster conversion rates. In addition, it is well-known that the utilization of ultrasound in electrochemical processes can offer many advantages, including electrode surface activation, degassing at the electrode surface, electrolyte degassing, disruption of the Nernst diffusion layer, and enhancement in mass transfer through the electrode double layer [24]. Therefore, we explore the effect of the ultrasonic treatment using an immersed ultrasound probe in this study. Ultrasonication is a high-energy density process, and the energies involved are sufficiently high to produce cavitation in liquids and thus can be used to influence chemistry and processing. Ultrasound is the mechanical wave created at a frequency of 20–106 kHz, a wavelength of 0.01–10 cm can reach a wave speed of ∼ 1500 m/s [25], [26]. While cavitation events in ultrasonic treatment can facilitate dispersion of initially aggregated CNTs in PVF/chloroform, ultra-sonification also increases the suspension temperature without any temperature control, a destabilizing factor for suspension. Consequently, we hypothesize that the duration and amplitude of the ultrasound should be optimized PVF/CNT electrodes production process. In the present study, we quantify how the PVF/CNT ratio, ultrasonic amplitude and duration alter the dispersion quality of the PVF/CNT, consequently, the maximum adsorption efficiency.

In addition to a high dispersion quality in the electrode preparation process, a large amount of active surface sites and a high adsorption efficiency are other desired properties of PVF/CNT electrodes for scale up of this selective separation process. To design PVF/CNT electrodes with these desired properties, a statistical model was used to optimize conditions for electrode fabrication, examine the effects of PVF/CNT and ultrasonication together, and better understand how co-effects influence the quality of the fabricated electrode. A response surface methodology (RSM) analyzes the relative significance of different operating parameters in complicated systems [27], [28], [29], [30], [31], [32]; therefore, we used this method to determine the optimal conditions for the preparation of the electrodes. A Box–Behnken design [33], a useful and efficient RSM method, was used to optimize the process variables. A new aspect of this work was to first investigate the variables that affect PVF/CNT dispersion quality and any possible interactions. The experimental design was used to find the optimal conditions resulting in the highest number of active surface sites and the greatest adsorption efficiency. To this end, PVF to CNT ratio, sonication duration, and ultrasonic amplitude were selected as independent variables. Moreover, the morphological and structural characteristics of the ultrasonically fabricated PVF/CNT electrodes were evaluated using X-ray diffraction (XRD), Raman spectroscopy, and scanning electron microscopy (SEM). The results of the optimization and characterization from the present study will provide useful guidance for fabricating the PVF/CNT electrodes with higher adsorption capacity essential for scale up.

## 2 Materials and methods

### 2.1 Materials

Polyvinyl ferrocene (PVF, CAS number 34801–99-5) was purchased from Polysciences. Multi-walled carbon nanotubes (CNTs, CAS number 308068–56-6), anhydrous chloroform (CAS number 67–66-3), potassium formate (CAS number 590–29-4), and lithium perchlorate (CAS number 7791–03-9) were obtained from Sigma Aldrich. Toray carbon paper (CP, TGP-H-60) was purchased from Alfa Aesar. Ultrapure water (Millipore MilliQ IQ 7000 system, 18 mΩ cm) was used to prepare the solutions.

### 2.2 Experimental methods

The experimental procedure was divided into five parts: 1) sonochemically preparing a combination of the PVF/CNT solutions at different ratios, sonication duration, and ultrasonic amplitude; 2) preparing the PVF/CNT electrodes using drop-casting methods; 3) characterizing in detail and measuring the electrochemically prepared electrodes; 4) investigating the adsorption efficiency of the fabricated electrode for formate ions; and 5) optimizing the conditions under which the highest adsorption capacity was observed.

#### 2.2.1 Sonochemical preparation of PVF/CNT solutions

First, six different solutions each using a different PVF/CNT mass ratio (0:1, 0.5:1, 1:1, 2:1, 3:1, and 1:0) were prepared. The dispersions were made as follows: the appropriate ratios were weighed (e.g., for a 1:1 ratio 40 mg PVF and 40 mg CNT were weighed) and mixed with 10 mL anhydrous chloroform in a 20-mL glass vial. A Cole-Parmer ultrasonic homogenizers, equipped with a 0.5-inch diameter immersion ultrasonic probe with timer and temperature controller, was used as the ultrasonic processor, working 20 kHz operating frequency. Second, the ultrasonic probe was lowered into the solution until the tip of the probe reached the middle of the solution without touching the vial; the vial was then sealed with parafilm and kept in an ice bath during sonication to keep the temperature constant and avoid extra heat generation. Hereafter, the sonication started. All mixtures prepared with different PVF/CNT ratios were individually sonicated for 1 h at 25 % amplitude at constant frequency of 10 s ON and 5 s OFF.

To investigate the sonication duration, and ultrasonic amplitude effects, the experiments were performed with a PVF/CNT 1:1 ratio and 25 % ultrasonic amplitude and a PVF/CNT 1:1 ratio and 1-h ultrasonic time, respectively.

#### 2.2.2 Electrode preparation and electrochemical measurements

Toray carbon paper (CP) was used to create the working electrodes using 1x 2 cm pieces cut as the surface of the working electrode, where the surface area used for drop-casting was 1 cm^2^. Immediately after preparing the PVF/CNT solutions, 50 μL solution was dropcasted onto one side of the electrodes for cyclic voltammetry (CV) measurements. The wet electrodes were then dried in an oven at 30 °C for 60 min. The dried electrodes were then used for the analysis. The CV measurements were performed using a three-electrode system in which the fabricated PVF/CNT electrode, a platinum plate, and Ag/AgCl were used as working electrode, counter electrode, and reference electrode, respectively. All CV experiments were recorded in 0.1 M LiClO_4_ solution by a Biologic SP-200 potentiostat at a scan rate of 5 mV/s under argon atmosphere at room temperature. Since the number of activated surface sites is directly related to the total charge passed due to the oxidation of ferrocene, the charge can be calculated by integrating the area under the anodic peak of a CV. The area was determined with the Biologic EC-lab peak-analysis tool. Eq. (1) shows the relation between the charge and the measured current:

(1) $$Q=\int_{t_{1}}^{t_{2}}\mathrm{Idt}=\frac{1}{\nu }\int_{E_{1}}^{E_{2}}\mathrm{IdE}$$

where *Q* is the charge (C), *v* is the sweep rate (V/s) and *E_1_* and *E_2_* are the potentials where the anodic peak starts and ends (V). The number of active surface sites can be determined from the charge as shown in the following Eq. (2):

(2) $$N_{\mathrm{sites}}=\frac{Q}{\mathrm{nF}}$$

where *N_sites_* is the number of activated surface sites in moles, *Q* is the calculated charge (C), *F* is the Faraday constant (C/mol), and *n is* the number of electrons transferred per reaction.

#### 2.2.3 Electrode characterization

X-ray diffraction (XRD) analysis was carried out using a Bruker D2 Phaser Tabletop Diffractometer with Cu Kα radiation (λ = 1.5418 Å) within the 2θ range of 5 to 60° to determine the structure of the fabricated electrodes. In addition, Raman spectra were collected using a JY Horiba LabRAM HR Raman spectrometer at room temperature between 1000 and 1800 cm^−1^ to provide more information on structure of the electrodes. The surface morphology of the fabricated electrodes was visualized using scanning electron microscopy (SEM, Jeol 6010LA) with an accelerating voltage of 15 kV.

#### 2.2.4 Adsorption and desorption

After the electrodes were characterized, the performance of the working electrodes with respect to formate adsorption and desorption was tested using chronoamperometry. The electrodes used for adsorption measurements differed slightly from those used for the CV measurements in terms of used solution volume. Similar with the previous studies in the literature [12], 200 μL solution was dropcasted onto the electrodes for adsorption and desorption experiments to make the amount of formate adsorbed more easily detectable. A potential of 0.6 V and 0.2 V vs Ag/AgCl was applied for 15 min for adsorption and desorption experiments, respectively. Eq. (3) was used to determine the amount of adsorbed formate:

(3) $$N_{\mathrm{ads}}=\left(C_{0}-C_{1}\right)\times V_{e}$$

The difference between the initial concentration (*C_0_*) and the concentration after adsorption (*C_1_*), multiplied by the electrolyte volume (*V_e_*) resulted in the amount of adsorbed formate (*N_ads_*) by the redox active material on the electrode surface. The concentrations were detected from the integrated areas of the peaks observed in HPLC (Agilent 1260 Infinity). Sample vials were placed in an autosampler and 10 µL was injected onto two Aminex HPX 87-H columns (Biorad) placed in series. During analysis the column oven temperature was maintained constant at 60 °C, with a steady flow of rate of 0.600 mL/min of an aqueous 1 mM H_2_SO_4_ eluent. Formate was detected using a RI detector with a temperature kept constant at 35 ⁰C.

#### 2.2.5 Experimental design and statistical analysis

RSM is a recognized statistical technique for identifying relationships among the effects of experimental variables in a study design. It includes a set of mathematical and numerical methods with benefits for modeling and analyzing problems with several variables that affect the response with the aim of optimizing the response. In the present study, the number of active surface sites on the electrode and the formate adsorption efficiency were the studied responses.

Using experimental design models helps to quickly conduct experiments, reduces the cost of testing, optimizes the effective parameters with a minimal number of experiments, and enables the investigation of the interaction among parameters. The present study used a three-factor, three-level BBD, which entailed a set of midpoints and a replicated central point in the multidimensional cube using 15 experiments, as computed by Design Expert (https://www.statease.com/software/design-expert/), and comprising 12 factorial points and 3 center points (Eq. (4)).

(4) $$N=2k\left(k-1\right)+C_{0}=2\times 3\left(3-1\right)+3=15$$

where *N* is the number of the experiments, *k* is the number of independent variables and *C_0_* is the replicate number of central point.

Each experiment was conducted twice. The PVF/CNT ratio (A), sonication duration (B), and ultrasonic amplitude (C) were the independent variables, each of which was analyzed as follows: −1, low level; +1, high level; and 0, midpoint to identify the experimental error value. The responses were the number of active surface sites and adsorption efficiency. The levels and ranges of variables used in this study are shown in Table 1.

**Table 1:** Range and levels of variables in Box–Behnken experimental design.

Design variable	Symbol	Range and levels		
		−1	0	1
PVF/CNT	A	1	2	3
Sonication Duration (h)	B	1	2	3
Ultrasonic Amplitude (%)	C	25	50	75

Following the experiments, we used a second-order polynomial regression model equation to reveal the relationship between the predicted response and the process variable. The general form of this equation is as follows:

(5) $$Y=\beta_{0}+\sum_{i=1}^{k}\beta_{i}X_{i}+\sum_{i=1}^{k}\beta_{\mathrm{ii}}X_{i}^{2}+\sum_{i_{i=1}}^{k}\sum_{j>1}^{k}\beta_{\mathrm{ij}}X_{i}X_{j}$$

where Y shows the predicted response, *β_0_* is the constant term, *β_i_* is the linear coefficient, *β_ii_* is the quadratic coefficient, and *β_ij_* is the interaction coefficient.

Analysis of variance (ANOVA) based on BBD was conducted using Design Expert (https://www.statease.com/software/design-expert/) to identify the fitness, suitability, and significance of the model coefficient. ANOVA is important for examining the significance and fit of the second-order polynomial equation. We used different statistical parameters, a lack of fit test, and multiple determination coefficients (*R^2^*) to determine the model significance. An F-test, which is used to compare statistical models fitted to a dataset to identify that shows the best fit, was also conducted to determine the significance of the effects.

## 3 Results and discussion

### 3.1 Electrochemical characterization

The performance of the fabricated PVF/CNT electrode was closely dependent on the dispersion of the PVF/CNT hybrids in chloroform. Better dispersion indicated that CNT was more stable in chloroform when PVF was present, which resulted from stabilizing π–π interactions between the PVF and CNT cyclopentadienyl rings, as described by Mao et al. [21]. To show the effect of ultrasonication on PVF/CNT electrode performance, the PVF/CNT electrodes prepared with and without ultrasonication were characterized using CV measurements. As shown in Fig. S1, ultrasound provides better dispersion results leading to more active surface sites on the PVF/CNT electrodes for formate adsorption; therefore, in addition to PVF/CNT, the sonication duration and ultrasonic amplitude were used to determine whether they influenced the dispersion quality and thus the number of active surface sites and formate adsorption efficiency. The fabricated PVF/CNT electrodes were characterized using CV measurements to determine the electrochemical stability (see Figs. S2-S4). Using the CV measurements, the performance of the electrodes was investigated and compared with the results of that from previous studies [12], [21]. As can be seen from Figs. S2-S4, the cycles of each electrode were very similar to each other, the peak area appeared to be close to constant, and the peak potentials of the different cycles remained similar. As explained before, the area under the anodic peak can be used to calculate the number of active surface sites on the electrode. Hence, the consistency of the anodic peak indicated that there was no loss in active sites, meaning that the fabricated electrodes were stable in the used electrolyte. Additionally, this shows that the redox reaction is chemically reversible, indicating that this separation method can be used multiple times without losing any performance and significant separation capacity per adsorption–desorption cycle. However, a comparison of CVs of different electrodes, fabricated using different conditions, showed significant differences. Comparing the areas under the anodic peaks of each electrode can be used to determine the best performing electrode.

**Fig. 1:**
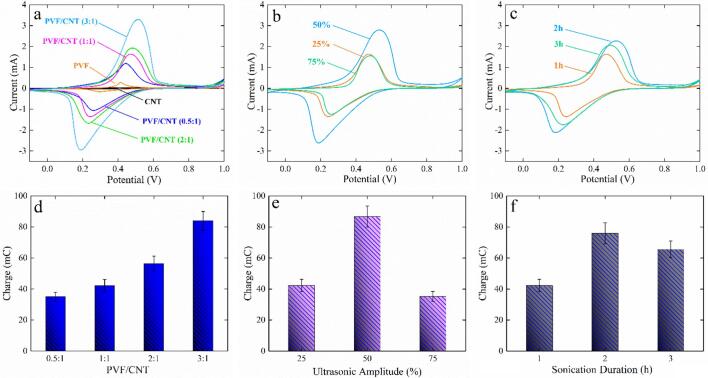
Cyclic voltammetry (CV) comparison of the PVF/CNT electrodes prepared at different (a) PVF/CNT ratio, (b) ultrasonic amplitude, and (c) sonication duration under Ar in 0.1 M LiClO4 at the scan rate of 5 mV/s. Electrochemical charge comparison of the PVF/CNT electrodes prepared at different (d) PVF/CNT, (e) ultrasonic amplitude, and (f) sonication duration.

To determine the relative performance of the fabricated electrodes, the CV measurements of all electrodes produced using different PVF/CNT, sonication duration, and ultrasonic amplitude were compared (Fig. 1).

When comparing the cycles of the electrodes, two main differences were observed. First, both the anodic and cathodic peak potentials show a shift depending on the preparation method and the ink composition; therefore, the difference between the oxidation and reduction peak potentials of each electrode also changed, which influenced the electrochemical reversibility of the redox reaction. When the difference between the peaks increased, the electron transfer rate was likely to change. Thus, some electrodes had a better electron transfer rate than others. Second, differences in the peak areas for both the anodic and the cathodic peaks are observed, meaning that the performance of the electrode is highly dependent on the ink composition and preparation method. Oxidized ferrocene units on the electrode surface selectively adsorb formate from the solution [12], [21]. Therefore, the number of active surface sites for formate adsorption is directly related to the number of oxidized ferrocene units on the surface and the area under the anodic peak in the CVs, as this displays the charge used to create oxidized ferrocene units on the surface.

The calculated charge of the oxidation peaks from CVs is proportional to the amount of PVF deposited. As can be seen in Fig. 1, the fabricated electrode with PVF/CNT values of 3:1 and 0.5:1 had the highest and lowest charges of 84.0 ± 6.0 and 34.97 ± 2.9 mC, respectively. In addition, differences in the preparation of the PVF/CNT dispersion led to significant differences in the amount of oxidized ferrocene units on the electrode surface. While an ultrasonic amplitude of 25 % and 75 % showed similar results, leading to charges of approximately 40 mC, electrodes fabricated with a dispersion prepared at an ultrasonic amplitude of 50 % performed much better, with a charge of 86.8 ± 6.8 mC. For the sonication duration an initial increase in the charge is observed at 2 h of sonication, which decreased with an increasing sonication duration to 3 h.

### 3.2 Experimental design results

A three-variable BBD was used to optimize the process of fabricating the electrode having the highest number of active surface sites and improving the efficiency of formate adsorption. The effects of PVF/CNT ratio, sonication duration, and ultrasonic amplitude on the number of electrode active surface sites and formate adsorption efficiency were evaluated using BBD to investigate the correlation between the combined effects of the individual parameters and both responses. The BBD matrix, experimental results from all the tested combinations of variables, and the corresponding responses for each run are provided in Table 2.

**Table 2:** Box-Behnken experimental design matrix and results.

Run order	Actual level of variables			Coded level of variables			Response	
	PVF/CNT	Sonication Duration	Ultrasonic Amplitude	A	B	C	Number of active surface sites (%)	Adsorption efficiency (%)
1	2	1	25	0	−1	−1	62.0	33.0
2	2	3	25	0	+1	−1	63.8	35.9
3	2	1	75	0	−1	+1	56.4	29.3
4	2	3	75	0	+1	+1	46.4	13.4
5	1	1	50	−1	−1	0	94.7	77.1
6	1	3	50	−1	+1	0	80.3	70.5
7	3	1	50	+1	−1	0	97.8	90.7
8	3	3	50	+1	+1	0	93.0	76.3
9	1	2	25	−1	0	−1	83.7	67.8
10	1	2	75	−1	0	+1	74.8	46.0
11	3	2	25	+1	0	−1	92.8	74.5
12	3	2	75	+1	0	+1	81.7	59.0
13	2	2	50	0	0	0	89.0	65.2
14	2	2	50	0	0	0	92.3	66.2
15	2	2	50	0	0	0	91.9	67.0

Eqs. (5), (6) are polynomial equations showing the empirical relationships between the responses and the independent variables regarding factors coded for both the number of active sites (NAS) and the adsorption efficiency (AE).

(6) $$Y_{\mathrm{NAS}}=91.07+3.97A-3.43B-5.38C+2.40AB-0.55AC-2.95BC+13.24A^{2}-12.86B^{2}-21.06C^{2}$$

(7) $$Y_{\mathrm{AE}}=66.13+4.89A-4.25B-7.94C-1.95AB+1.57AC-4.70BC+23.22A^{2}-10.70B^{2}-27.53C^{2}$$

where *A* is PVF/CNT, *B* is the sonication duration, and *C* is the ultrasonic amplitude. While the highest number of electrode active surface sites (97.8 %) obtained at PVF/CNT ratio 3, 1 h ultrasonic duration, and 50 % ultrasonic amplitude was in run 7, the lowest adsorption efficiency (13.4 %) was achieved at PVF/CNT 2, 3 h ultrasonic duration, and 75 % ultrasonic amplitude in run 4. For both responses, the experimental data were statistically analyzed by ANOVA, and the results are presented in Table 3, Table 4.

**Table 3:** ANOVA results of the response surface quadratic model for number of active surface sites (%).

Source	Sum of Squares	df	Mean Square	F-Value	p-value Prob > F
Model	3532.97	9	392.55	86.31	< 0.0001
A-PVF/CNT	126.40	1	126.40	27.79	0.0033
B- Sonication Duration	93.85	1	93.85	20.63	0.0062
C-Ultrasonic Amplitude	231.13	1	231.13	50.82	0.0008
AB	23.04	1	23.04	5.07	0.0742
AC	1.21	1	1.21	0.27	0.6280
BC	34.81	1	34.81	7.65	0.0395
A^2^	647.42	1	647.42	142.34	< 0.0001
B^2^	610.47	1	610.47	134.22	< 0.0001
*C*^2^	1637.37	1	1637.37	359.99	< 0.0001
Residual	22.74	5	4.55	–	–
Lack of Fit	16.26	3	5.42	1.67	0.3957
R^2^	0.9936				

Adj R^2^ = 0.9821, Pred R^2^ = 0.9228, Adequate Precision = 29.317, Standard Deviation = 2.13.

**Table 4:** ANOVA results of the response surface quadratic model for adsorption efficiency (%).

Source	Sum of Squares	df	Mean Square	F-Value	p-value Prob > F
Model	6539.59	9	726.62	145.10	< 0.0001
A-PVF/CNT	191.10	1	191.10	38.16	0.0016
B- Sonication Duration	144.50	1	144.50	28.85	0.0030
C-Ultrasonic Amplitude	504.03	1	504.03	100.65	0.0002
AB	15.21	1	15.21	3.04	0.1418
AC	9.92	1	9.92	1.98	0.2183
BC	88.36	1	88.36	17.64	0.0085
A^2^	1990.92	1	1990.92	397.56	< 0.0001
B^2^	423.06	1	423.06	84.48	0.0003
*C*^2^	2798.23	1	2798.23	558.77	< 0.0001
Residual	25.04	5	5.01		
Lack of Fit	23.41	3	7.80	9.60	0.0958
R^2^	0.9962				

Adj R^2^ = 0.9893, Pred R^2^ = 0.9424, Adequate Precision = 43.086, Standard Deviation = 2.24.

ANOVA for both responses showed that the results of the models were statistically significant because the models’ F-values were 86.31 and 145.10, and the corresponding p-values were < 0.0001, which indicated only a 0.01 % chance of the F-value occurring because of noise.

In addition, the high *R^2^* values, a measure of the model’s degree of fit, were 0.9936 and 0.9962, which indicated good correlation between the measured and predicted response values. The ‘Pred R^2^′ value was reasonably in accordance with the “Adj R^2^” value. Adeq Precision was used for measuring the signal to noise ratio, which is considered desirable if it is higher than 4. In this study, the signal to noise ratio was determined as >4. Thus, the quadratic model could be used to navigate the design space.

According to Table 3, Table 4, the independent parameters A, B, and C; the interaction between sonication duration and ultrasonic amplitude (BC); and the quadratic terms A^2^, B^2^, and C^2^ were highly significant parameters for both responses with *p*-values < 0.05; however, the remaining terms showed no significant impact. The order in which the independent variables affected the number of active surface sites and formate adsorption efficiency were C > A > B. Among these, ultrasonic amplitude (i.e., C) was the major factor that affected both responses.

**Fig. 2:**
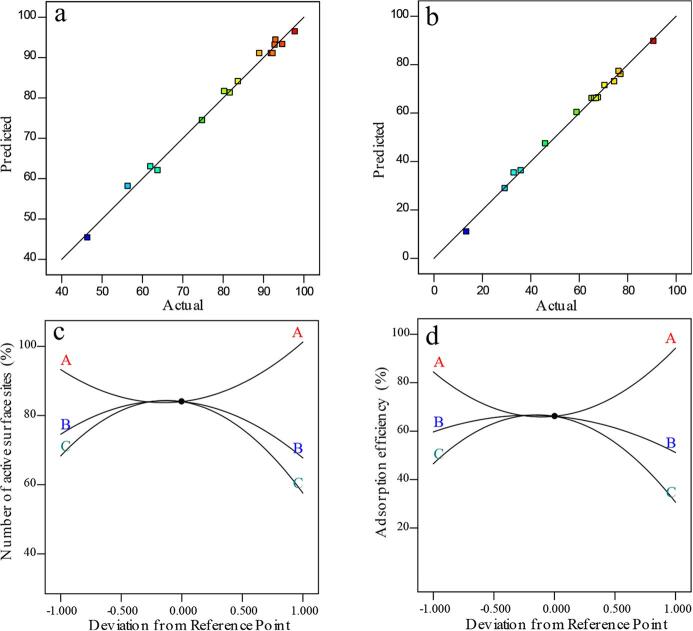
Plot of the predicted response versus observed response for (a) the number of active surface sites (%), and (b) formate adsorption efficiency (%). The perturbation plot of each factor (A: PVF/CNT ratio, B: sonication duration, and C: ultrasonic amplitude) for (c) the number of active surface sites (%), and (d) formate adsorption efficiency (%).

The relationship between the predicted and actual values for the number of active surface sites and formate adsorption efficiency is provided in Fig. 2.

Fig. 2 clearly presents that the predicted values are close to the observed experimental values, which meant that BBD was suitable for both responses. Fig. 2 also shows the perturbation plots used to examine the simultaneous effect of three factors on formate adsorption efficiency. This plot determined formate adsorption efficiency as each variable moved from the preferred reference point; all other factors were held constant at zero. Hence, the perturbation plot exhibits the deviation of the factorial level from the adjusted reference point of all the variables. The abscissa shows the deviations from reference point ranging from −1 to +1 as the coded value and the ordinate standard for the change of formate adsorption efficiency based on the influence of a single factor. The sharp curvature seen in each of the variables (i.e., A, B, and C) indicates that they had a significant effect on maximum formate adsorption efficiency; however, the effect of the positive and negative deviations was different. For the effects of sonication duration and ultrasonic amplitude, when the deviations ranged from −1 to +1, the formate adsorption efficiency first increased and then decreased. This reaction was the opposite of that for PVF/CNT ratio; therefore, when the values of PVF/CNT and sonication duration were moderate, but ultrasonic amplitude ratio was high, the fabricated electrode resulted in maximum formate adsorption efficiency.

**Fig. 3:**
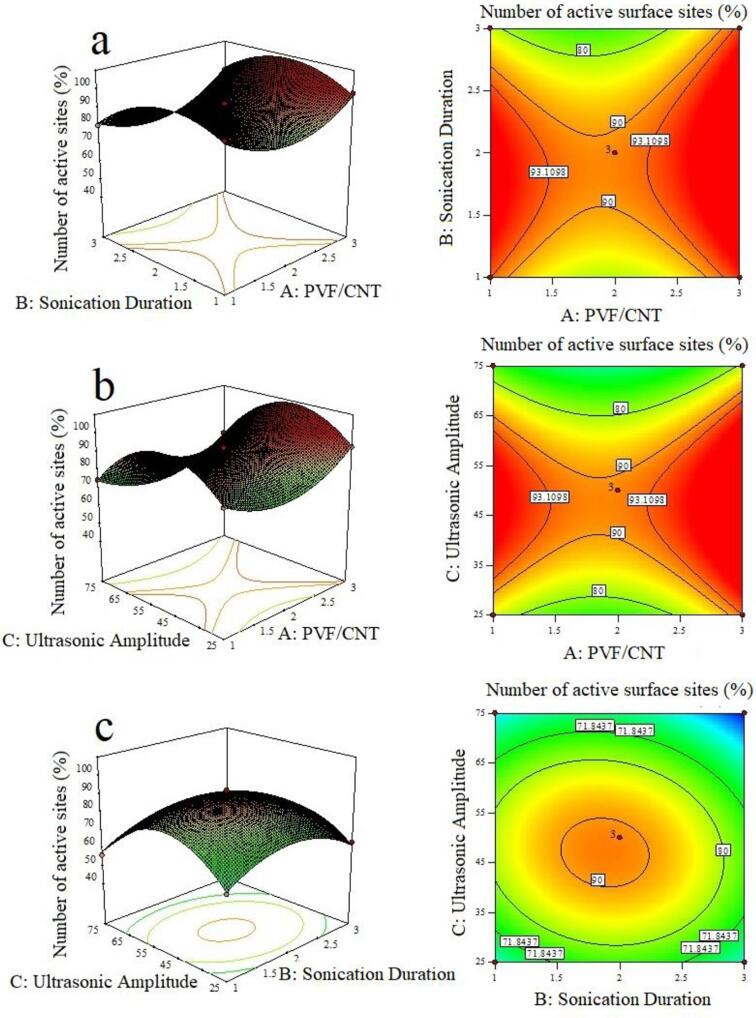
Three-dimensional (3D) response surface plots and two-D (2D) contour plots showing the effects of (a) PVF/CNT and sonication duration, (b) PVF/CNT and ultrasonic amplitude, and (c) sonication duration and ultrasonic amplitude on the number of active surface sites (%).

**Fig. 4:**
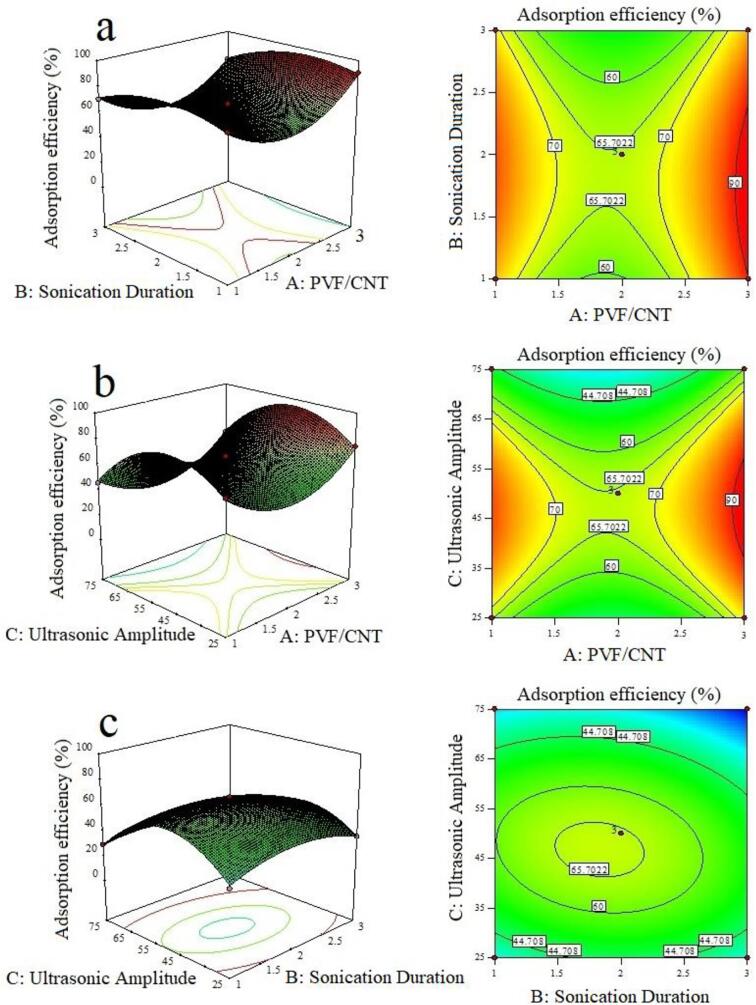
Three-dimensional (3D) response surface plots and two-D (2D) contour plots showing the effects of (a) PVF/CNT and sonication duration, (b) PVF/CNT and ultrasonic amplitude, and (c) sonication duration and ultrasonic amplitude on formate adsorption efficiency (%).

Using RSM, the effects of the independent variables and their interaction on formate adsorption efficiency and surface site variables were graphically displayed by both three-dimensional (3D) response surface plots and two-D (2D) contour plots. These plots represent a graphical depiction of the refined quadratic equations obtained after reducing the model, which demonstrated the relationship between the effects of the experimental variables and the responses for optimized conditions. The 3D plots are a function of two factors when all other parameters are maintained at fixed levels; they are helpful for understanding the effect of the two interacting factors. The effects of PVF/CNT and sonication duration (a), PVF/CNT and ultrasonic amplitude (b), and sonication duration and ultrasonic amplitude (c) on the number of active surface site and adsorption efficiency are given in Fig. 3, Fig. 4, respectively.

It is clear that the adsorption efficiency of the fabricated electrodes is sensitive to all three variables. Considering the 3D and 2D plots together, a desirable high response value will be achieved with a higher PVF/CNT ratios and lower sonication durations. Under low and medium ultrasonic amplitudes, an increase in PVF/CNT positively affects both responses. That is, the number of active surface sites on the electrodes and the formate adsorption efficiency simultaneously increases. Increasing the sonication duration at low ultrasonic amplitude (25 %) results in an increase in the number of active surface sites on the electrode, and thus, an increased efficiency of formate ion adsorption. With the increase in the ultrasonic amplitude value to 75 %, both responses were negatively influenced, and both the number of active surface sites and formate adsorption efficiency significantly decreases. Hence, operating at high amplitude and prolonged sonication duration negatively affected formate adsorption efficiency. Moreover, of the three variables studied, the most effective was the ultrasonic amplitude.

### 3.3 Morphological and structural characterization

The surface morphology of the prepared electrodes at different PVF/CNT values was elucidated using SEM. Fig. S5a–c show the SEM images of the untreated CP electrode and the CP electrode treated by electrochemical oxidation in the CNT/chloroform dispersion and PVF/chloroform dispersion, respectively. As shown in Fig. S5a, CP was woven with carbon fibers at a diameter of ∼10 μm. A fiber morphology nearly similar to that of the untreated CP electrode was observed for the CP electrode after oxidation in the CNT/chloroform dispersion (Fig. S5b). As stated in the literature, this indicated that CNTs alone could not be deposited onto the electrode [12], [21]. Comparing Fig. S5c with Fig. S5a and S5b indicates that the non-homogenous and uneven polymer layers formed on the electrode surface and partially filled the empty voids between carbon fibers after precipitation of PVF^+^. This was expected because once the polymer reached the surface of the electrode and was oxidized, it became solvophobic. The presence of PVF on the electrode surface was also confirmed by CV. As electrochemical oxidation was applied to PVF/CNT dispersion, it was crucial to evaluate whether CNTs and their polymer shells were deposited together. Because CNTs could not be deposited without PVF layers, if PVF was detached during oxidation, the resulting electrode surface would not include CNTs and the successful co-deposition of CNTs and PVF would suggest stable attachment of PVF to CNTs during redox transformation.

**Fig. 5:**
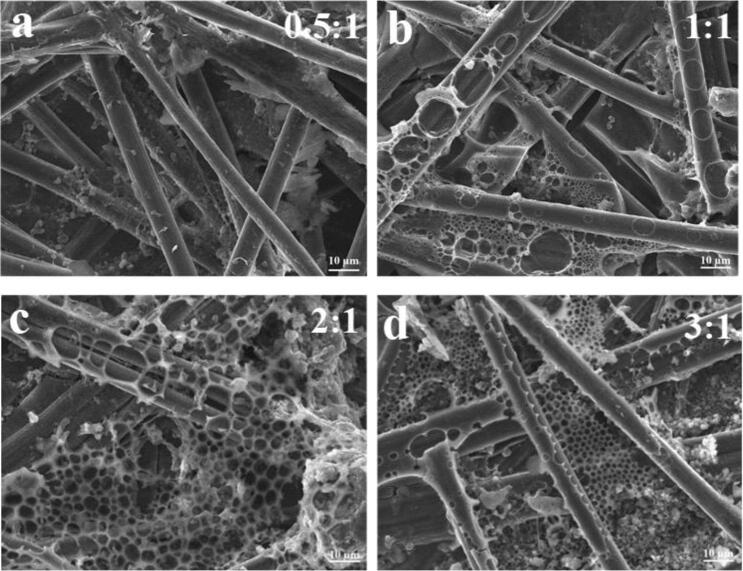
Effect of PVF/CNT ratio on the surface morphology of the electrodes obtained treated by electrochemical oxidation. Scanning electron microscopy (SEM) images of the electrodes fabricated at different PVF/CNT value. (a) 0.5:1, (b) 1:1, (c) 2:1, (d) 3:1.

Fig. S6a–b show the SEM images of the electrodes prepared without ultrasonication at two different PVF/CNT values. The SEM image in Fig. S6a illustrates the surface morphology of the electrode prepared in the absence of ultrasonic irradiation at a 1:1 ratio that appeared in the form of large, rounded, compact, irregular agglomerates. The SEM image in Fig. S6b shows that increasing the amount of PVF in the dispersion media without ultrasonication led to the variations in amount of the rough agglomerated particles on the CP surface. In addition, the coating on the surface of the electrode obtained without ultrasonic irradiation was not homogeneous.

**Fig. 6:**
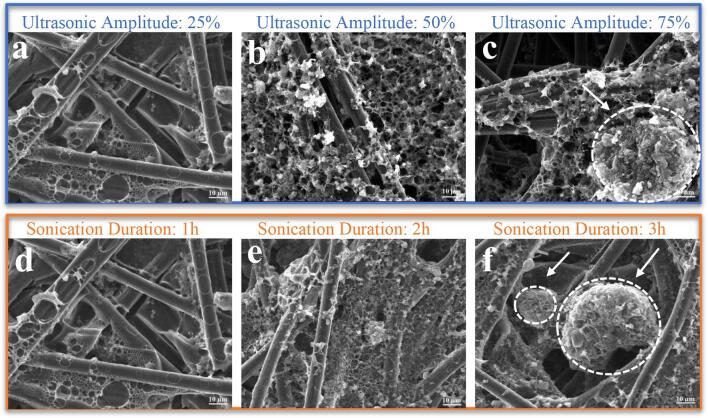
Effects of different ultrasonic treatments on the surface morphology of the PVF/CNT electrodes obtained treated by electrochemical oxidation. Scanning electron microscopy (SEM) images of the electrodes exposed for (a) 25 %, (b) 50 %, (c) 75 % ultrasonic amplitude at PVF/CNT 1 and 1-h ultrasonic time; (d) 1 h, (e) 2 h, (f) 3 h ultrasonic time at PVF/CNT 1 and 25 % ultrasonic amplitude.

Fig. 5a–d show the SEM images of the electrodes that were prepared with ultrasonication at four different PVF/CNT values.

First, the films that formed on the surfaces of these electrodes after being subjected to electrochemical oxidation were highly different from the pure polymer film seen in Fig. S5c. Significant changes occur in the surface morphology of the electrodes based on their PVF/CNT values and using ultrasonication. The lowest PVF/CNT value, namely 0.5:1 ratio, affected the surface morphology of the electrodes least, with little observed change. Moreover, the electrodes’ surface morphology was similar to that obtained in the CNT/chloroform dispersion medium. In other words, the morphology of the electrodes prepared at 0.5:1 ratio was similar to electrodes dropcasted by suspension consisting only of unmodified CNTs, which indicated a very low extent of polymer functionalization on CNTs in this hybrid. When the PVF/CNT ratio was highest, the surface morphology of the electrodes (Fig. 5d) partially resembled that of the surface obtained in the PVF/chloroform dispersion medium. The SEM images of the electrodes obtained at PVF/CNT values of 1:1, 2:1, and 3:1 clearly displayed the interconnected CNTs dispersed throughout the polymer film. When PVF/CNT was 1:1, a partially porous, random, and rough coating was observed around the carbon fiber. At PVF/CNT 2:1 and 3:1, interconnected micropores with a 3D framework surrounded by a conformal polymer coating was observed. The π-π interaction between ferrocene moieties and CNTs created a stable and dispersed film on the substrate, which allowed electrons to quickly migrate from the carbon fiber substrate to the redox-active center of the polymer [12], [21]. In addition, ionic transport within the electrode was facilitated by the porous structure of the polymer coating.

With a PVF/CNT ratio kept constant at 1:1 and a ultrasonication duration of 1 h, SEM analysis was performed to determine the effects of the ultrasonic amplitude on the surface morphologies of the prepared electrodes (Fig. 6a–c).

The surface morphologies of all three electrodes prepared under the 25, 50, and 75 % ultrasonic amplitude were distinct from each other. The increase in amplitude from 25 to 50 % resulted in the electrode surface being coated with a more homogeneous and porous structure, and the tendency for agglomeration on the electrode surface was little to none. Raising the ultrasonic amplitude value to 75 % resulted in the formation of significant amounts of large, tightly interconnected agglomerates on the electrode surface. That is, the coating on the surface was not homogeneous, and clusters of sphere-like agglomerations were observed on the surface. This agglomeration tendency showed that the 75 % ultrasonic amplitude value that was applied was not a suitable condition for the preparation of electrode solutions and caused a reduction in the dispersion quality. We hypothesize that the aggregation at high ultrasonication intensities can be triggered by either local heating of suspension and/or violent cavitation events. Despite the fact that the suspension was placed in an ice bath during ultra-sonification in this study, the local temperature increase in the vicinity of ultrasound probe could still lead to enhanced aggregation. Such temperature and cavitation induced destabilization of emulsions and suspension has been previously reported [34].

The effects of the ultrasonication duration on the surface morphologies of the prepared electrodes were similar to those of the amplitude parameter in that a severe tendency for agglomeration was observed on the surface morphology of the electrodes obtained under the longest ultrasonication duration (Fig. 6f). At 2 h ultrasonication duration, the surface morphology showed a significant improvement and a more homogeneous coating over that of 1 h ultrasonication.

**Fig. 7:**
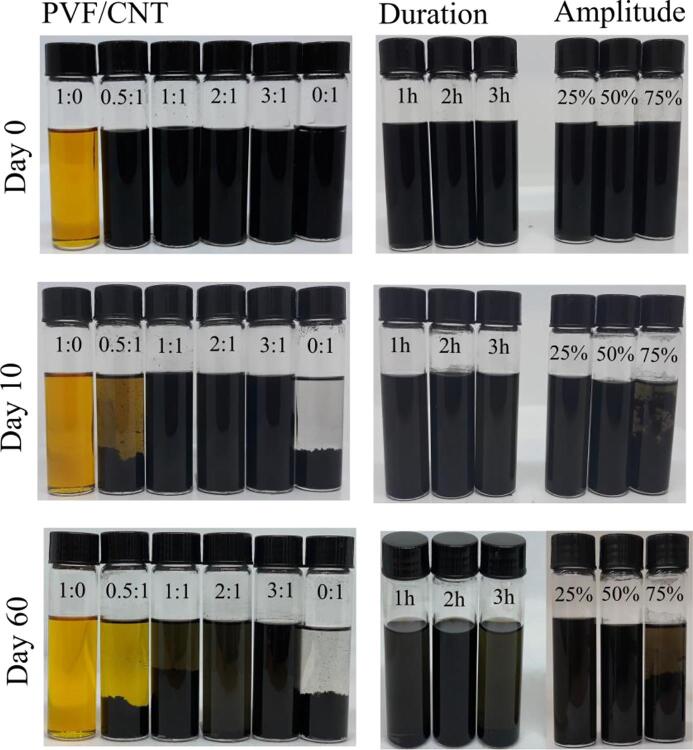
Effect of PVF/CNT, sonication duration, and ultrasonic amplitude on the sedimentation behaviors of the prepared electrode solutions. Photographs of the PVF/CNT dispersions in chloroform on day 0, day 10, and day 60.

Fig. 6d–f show that ultrasonication duration up to 2 h resulted in more homogeneous coating of PVF/CNT suspension of carbon electrode; however, longer durations resulted in irregularities on the electrode surface. The agglomerates observed in SEM images for both high ultrasonic amplitude and ultrasonication durations studied points out that the prepared suspensions stability could be influenced by these parameters. The suspensions could aggregate before or during drop-casting; therefore, the sedimentation behavior of the electrode suspensions prepared under different conditions were examined as a function of time. The suspension prepared in the absence of ultrasonic irradiation was very unstable and sedimented within <2 h. The sedimentation results obtained with ultrasonication are shown in Fig. 7. For suspensions with varying PVF/CNT ratio, the suspension with 3:1 ratio was stable even after 60 days. As the SEM images of 3:1 PVF/CNT ratio given in Fig. 5d shows the most conformal coating as well as the highest adsorption efficiency at fixed ultrasonication amplitude and duration, we conclude that the stability of suspensions is intimately connected to structure of PVF/CNT coating ultimately to adsorption efficiency. Suspensions with PVF/CNT ratio 0.5:1 and 0:1 sedimented within 10 days indicating that there was not enough PVF to stabilize CNTs in 0.5:1 sample. The dispersions sonicated with an amplitude of 25 and 75 % sedimented clearly, while the dispersion sonicated with an amplitude of 50 % showed no signs of sedimentation even after 60 days, which indicated that an ultrasonic amplitude of 50 % ensured a better dispersion stability at 1:1 PVF/CNT and 1 h sonication time. Moreover, we observed that suspensions that sedimented quickly corresponded to the electrode preparation conditions where high tendency of agglomeration is observed in SEM images. This result supports our hypothesis connecting suspension stability to electrode structure and adsorption efficiency. In other words, less stable suspensions observed to sediment quickly in Fig. 7 produce electrodes with aggregates on electrode surface hence and lower adsorption efficiency.

**Fig. 8:**
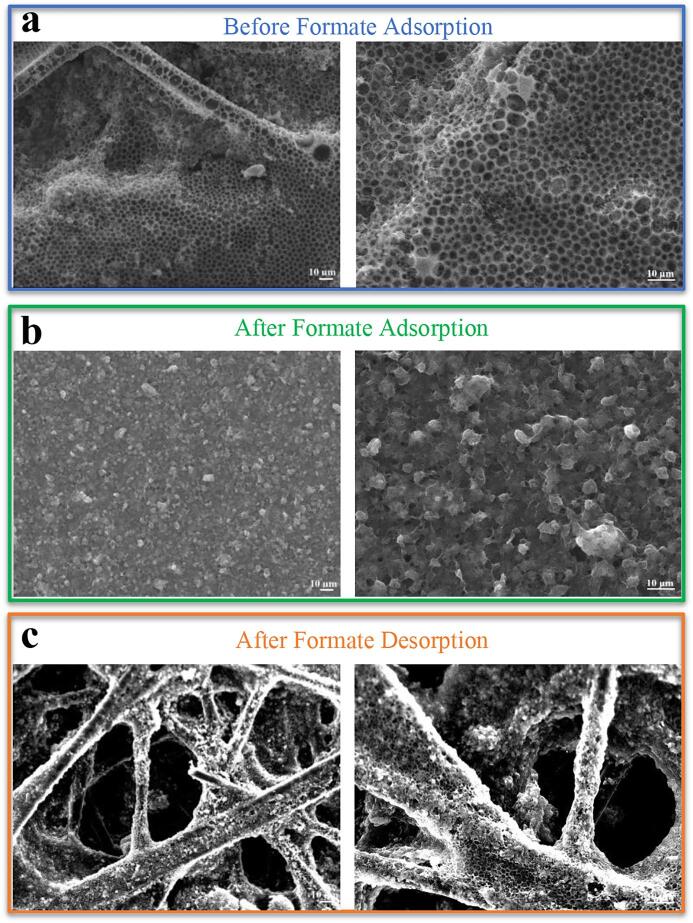
Scanning electron microscopy (SEM) images of the PVF/CNT electrodes obtained treated by electrochemical oxidation (a) before formate adsorption, (b) after formate adsorption, (c) after formate desorption.

Fig. 8a presents the SEM image of the electrode that was prepared under the conditions in which the maximum active surface sites and maximum adsorption capacities were obtained based on the experimental design results. Adsorption properties were greatly dependent on the morphology of the prepared electrode surface. The electrode surface had to be highly porous for the ions in the electrolyte to diffuse and interact with the material. When compared to the other SEM images, that in Fig. 8a shows a homogeneous, porous surface consisting of pores of nearly the same size. This fabricated electrode with a porous structure could provide easy access for ions at the interface between the active material and electrolyte and reduce the diffusion path and enhance the electrochemical performance of the electrode. The SEM images obtained after the adsorption and desorption of the formate ions onto the surface of the electrode prepared under these conditions are shown in Fig. 8b–c, respectively. It was morphologically determined that after the adsorption process, the pores on the electrode surface were completely filled (i.e., the formate ions were uniformly adsorbed onto the surface).

One of the interesting qualities of the redox electrodes is the reversibility of adsorption and the recovery of adsorbed compounds. This reversibility was tested with desorption experiments and determining the efficiency formate ion recovery. After the desorption process, we observed that large hollow pores reappeared on the electrode surface. Accordingly, the experimental design results were morphologically supported by SEM analysis and proved that the adsorption/desorption process was successful.

### 3.4 XRD and Raman analyses

In addition to morphological characterization, XRD and Raman analyses were conducted to structurally characterize the fabricated electrodes, and the results are shown in Fig. S7. The prominent diffraction peak for all electrodes prepared using different PVF/CNT ratios, sonication durations, and ultrasonic irradiation conditions was detected at 2θ = ∼26°, which was the result of the (0 0 2) graphite facet of the CP [35], [36]. The intensity and position of the major characteristic XRD peak of the fabricated electrodes in the XRD spectra was largely similar to each other. Typical peaks of PVF were not found, which could be the result of the production of thin film layers on the electrode surface and the dominant effect of CP peaks. That is, the structure of the fabricated electrodes was not destroyed during different experimental conditions. XRD analysis results were supported with Raman results. In the Raman spectra shown in Fig. S7b, there were two main peaks at ∼1350 cm^−1^ and 1580 cm^−1^ corresponding to D and G peaks respectively. The results were consistent with those reported from earlier studies [21], [37], [38], [39].

## 4 Conclusion

Electrochemical separation of formate, the smallest carboxylate, has been heavily investigated because the separation challenges in the recovery of carboxylate products formed during electroreduction of CO_2_, and in the current chemical and pharmaceutical industries. The aim of the present study was to use BBD to optimize the fabrication process of PVF/CNT based electrodes for formate separation for a higher separation efficiency. PVF/CNT based electrodes were fabricated using a dispersion prepared with ultrasound. The major role of ultrasonication is to provide a homogeneous PVF/CNT suspension for drop casting, consequently, contributing to homogeneous distribution PVF & CNT on the carbon electrodes’ surface. The characterization results showed that the ultrasonic amplitude and sonication duration significantly affected PVF/CNT dispersion. SEM images showed that the use of ultrasound negatively affected the morphology of the fabricated electrodes when irradiated for longer periods and at higher ultrasonic amplitude. This result was also supported by the experimental design analysis. The experimental values under optimal conditions (PVF/CNT ratio 3, 1-h ultrasonic time, and 50 % ultrasonic amplitude) agreed with the predicted values. BBD based on a second-order polynomial model was adequate for optimizing the ultrasound-assisted fabrication of PVF/CNT electrodes to selectively adsorb formate based on satisfactory ANOVA results and descriptive statistics parameters. Ultrasonic amplitude was identified as the most effective of the studied parameters dictating adsorption efficiency based on both responses. The PVF/CNT electrodes fabricated under these optimum conditions exhibited significantly higher formate adsorption efficiency. Based on electrode characterization and sedimentation study, we conclude that the ultrasound under optimum conditions produced more stable PVF/CNT dispersion, which favored the higher number of active surface sites on the fabricated electrodes and greater formate adsorption efficiency. We hope the ultrasound intensified electrode production process we systematically optimized in this study enables rational design of electrodes for electrochemical separations and beyond.

## CRediT authorship contribution statement

**Sevgi Polat:** Conceptualization, Methodology, Investigation, Validation, Visualization, Writing – original draft, Writing – review & editing. **Ruud Kortlever:** Conceptualization, Resources, Writing – review & editing, Supervision. **Huseyin Burak Eral:** Conceptualization, Resources, Visualization, Writing – review & editing, Supervision, Project administration.

## Declaration of Competing Interest

The authors declare that they have no known competing financial interests or personal relationships that could have appeared to influence the work reported in this paper.

## Data Availability

Data will be made available on request.
