# Supplementary material for: Ultrasound-promoted preparation of polyvinyl ferrocene-based electrodes for selective formate separation: Experimental design and optimization
Source: Ultrason Sonochem. 2022 Aug 30;89:106146. doi: 10.1016/j.ultsonch.2022.106146 (PMC9465433; doi:10.1016/j.ultsonch.2022.106146)
Supplement: Supplementary data 1 [file mmc1.pdf]

Supporting Information

Ultrasound-promoted preparation of polyvinyl ferrocene-based electrodes for selective formate separation: Experimental design and optimization

Sevgi Polat [a,b*], Ruud Kortlever [c], Huseyin Burak Eral [a*]

[a] Complex Fluid Processing section, Process & Energy Department, Faculty of Mechanical, Maritime and Materials Engineering, Delft University of Technology, 2628 CB Delft, The Netherlands

[b] Chemical Engineering Department, Faculty of Engineering, Marmara University, 34854 İstanbul, Turkey.

[c] Large-Scale Energy Storage section, Process & Energy Department, Faculty of Mechanical, Maritime and Materials Engineering, Delft University of Technology, 2628 CB Delft, The Netherlands

Corresponding authors: S.Polat@tudelft.nl, H.B.Eral@tudelft.nl

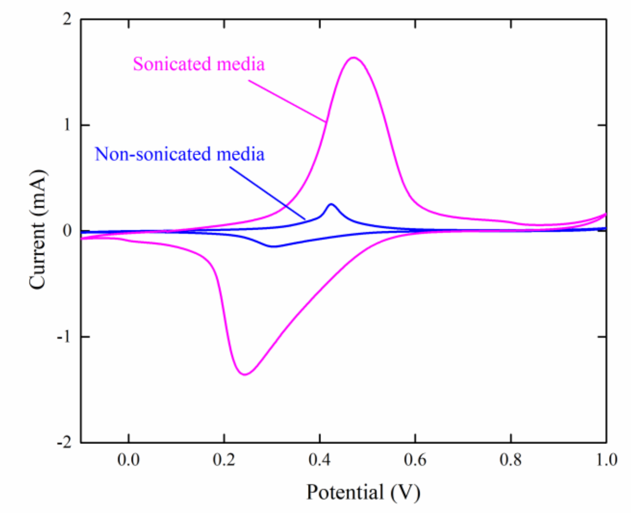

Figure S1. Cyclic voltammetry (CV) comparison of the PVF/CNT electrodes prepared at 1:1 PVF/CNT ratio with and without ultrasonication under Ar in 0.1 M $LiClO_4$ at the scan rate of 5 mV/s.

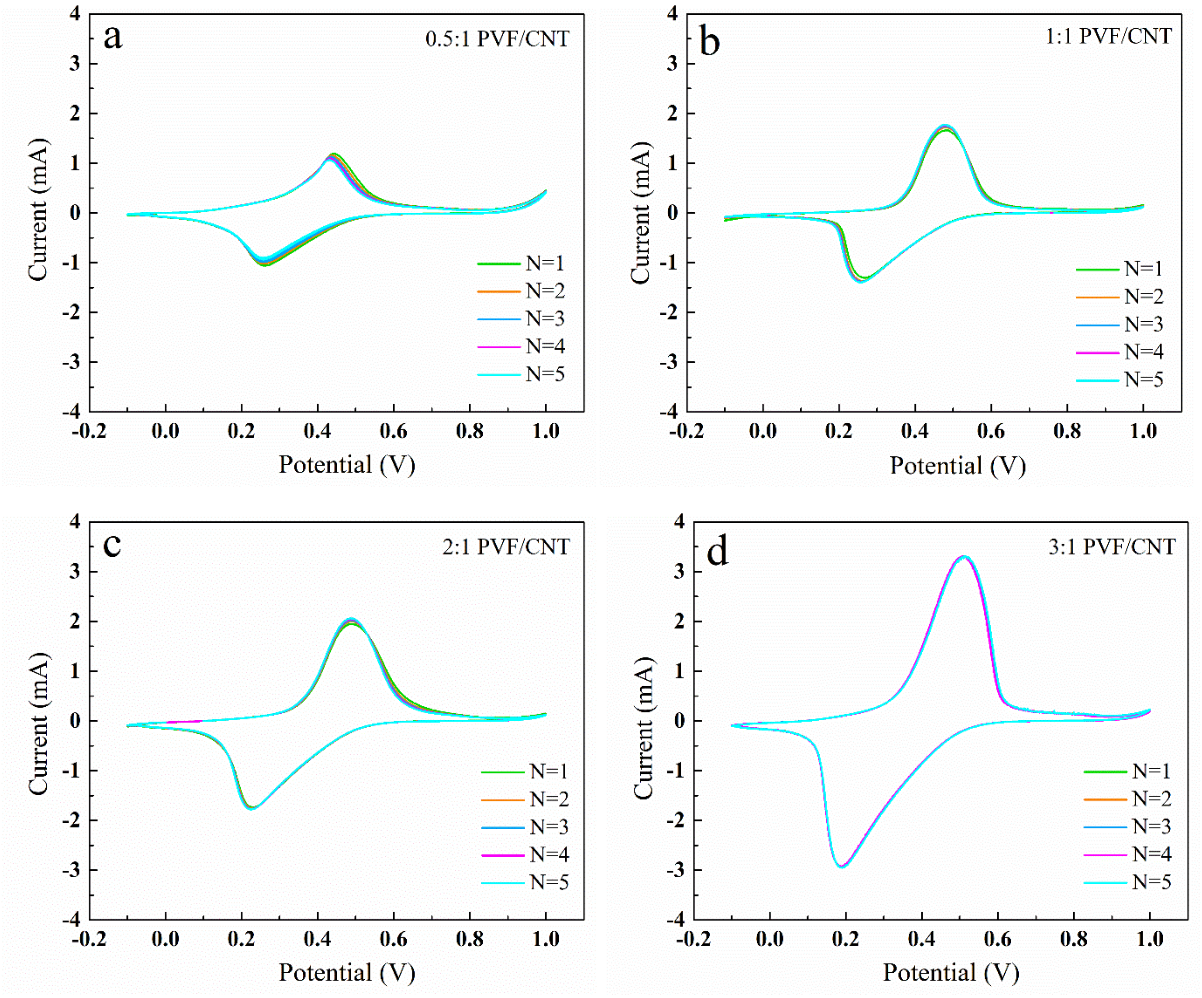

Figure S2. Cyclic voltammetry (CV) results of the PVF/CNT electrodes prepared at different PVF/CNT under Ar in 0.1 M $LiClO_4$ at the scan rate of 5 mV/s. (a) 0.5:1, (b) 1:1, (c) 2:1, and (d) 3:1. (*N* denotes the number of the potential cycles)

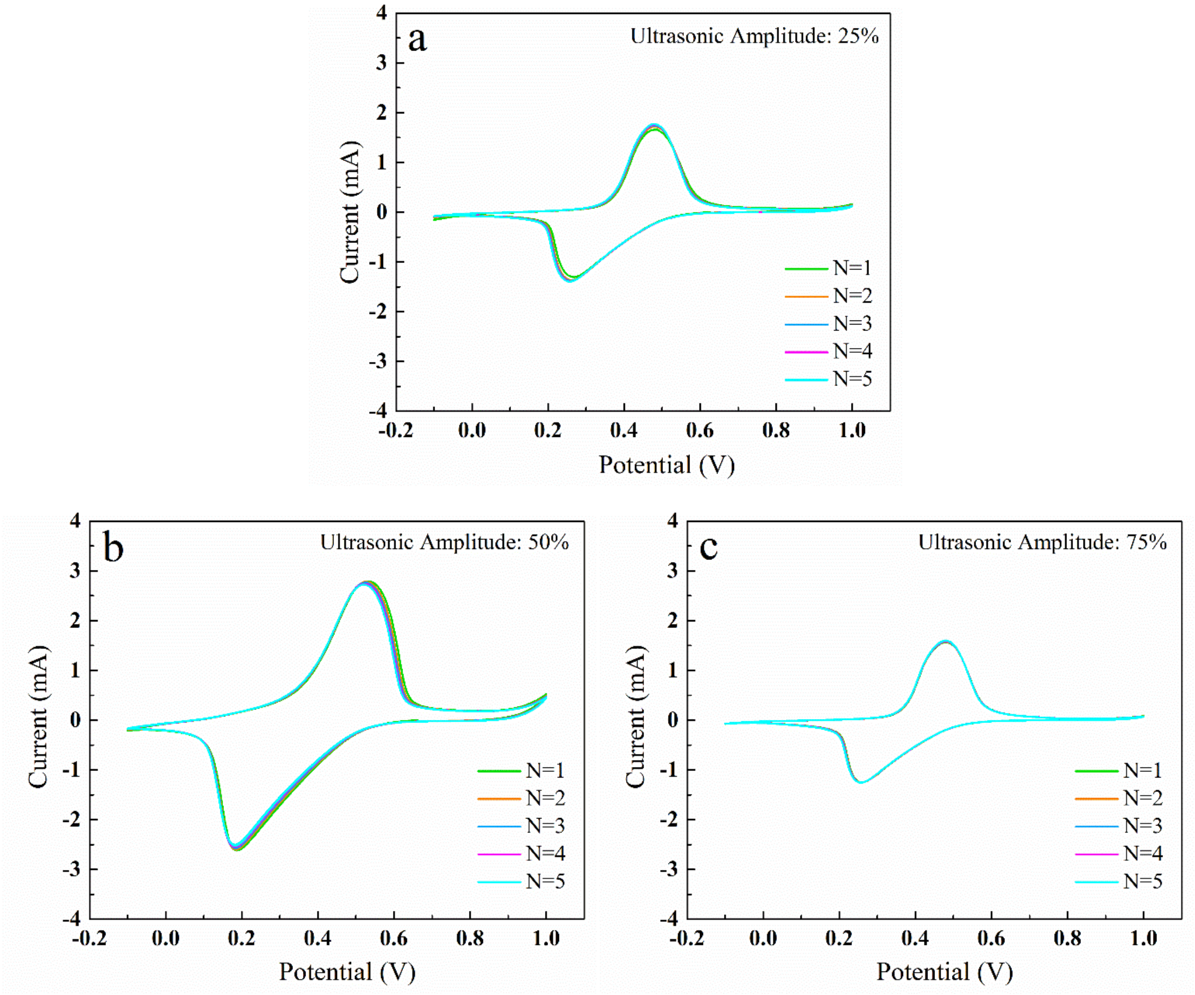

Figure S3. Cyclic voltammetry (CV) results of the PVF/CNT electrodes prepared at different ultrasonic amplitude under Ar in 0.1 M $LiClO_4$ at the scan rate of 5 mV/s. (a) 25%, (b) 50%, and (c) 75%. (*N* denotes the number of the potential cycles)

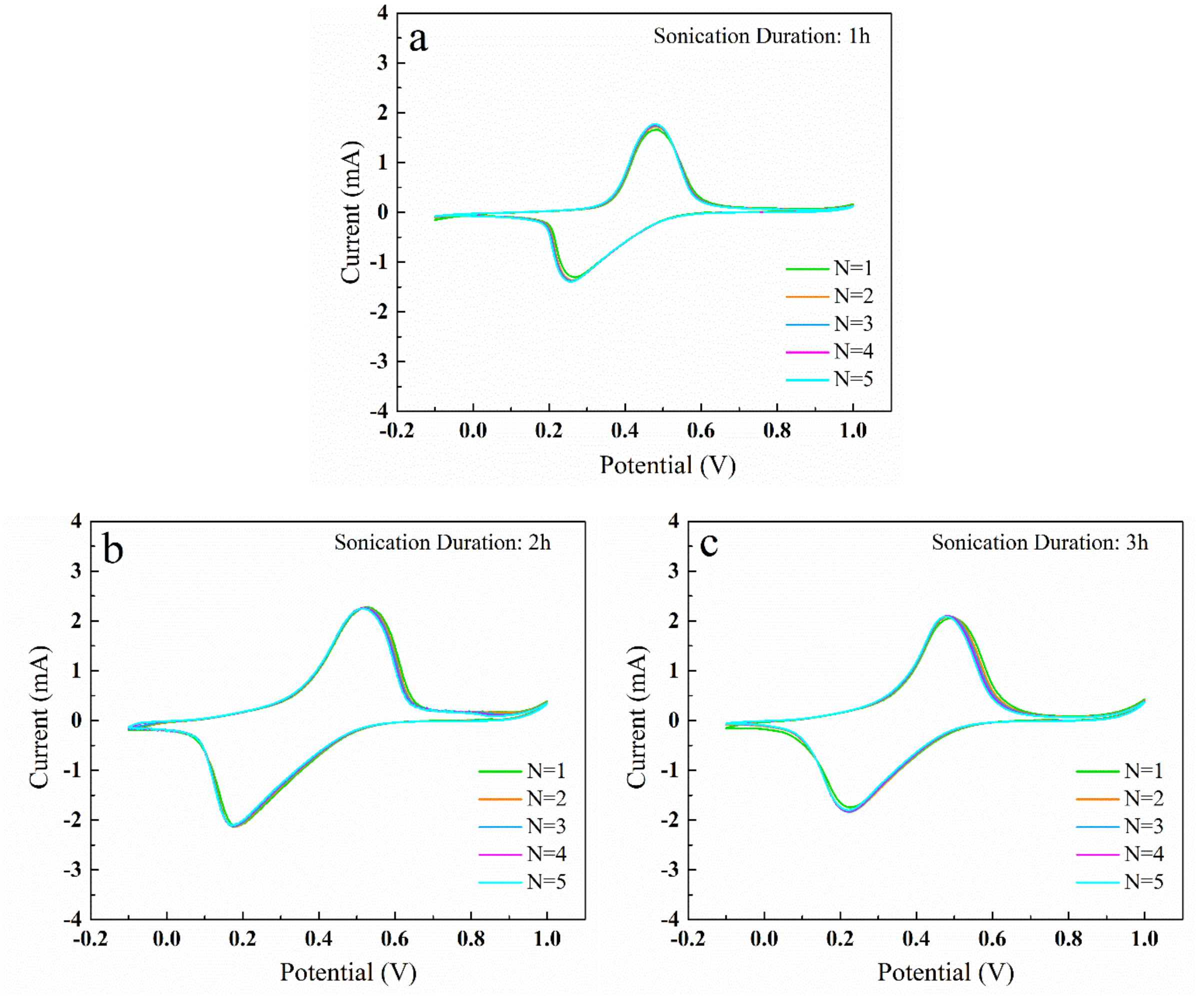

Figure S4. Cyclic voltammetry (CV) results of the PVF/CNT electrodes prepared at different sonication duration under Ar in 0.1 M $LiClO_4$ at the scan rate of 5 mV/s. (a) 1h, (b) 2h, and (c) 3h. (*N* denotes the number of the potential cycles)

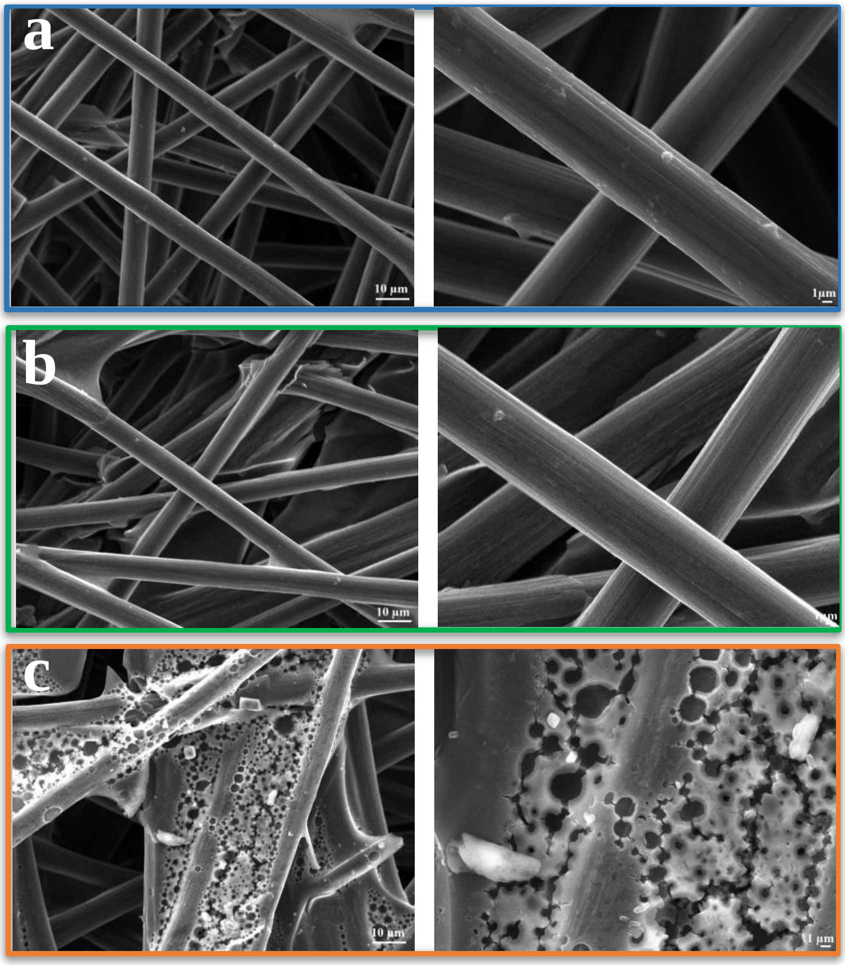

Figure S5. Scanning electron microscopy (SEM) images of (a) untreated carbon paper electrode, (b) carbon paper electrode treated by electrochemical oxidation in the CNT/chloroform dispersion, and (c) PVF/chloroform dispersion.

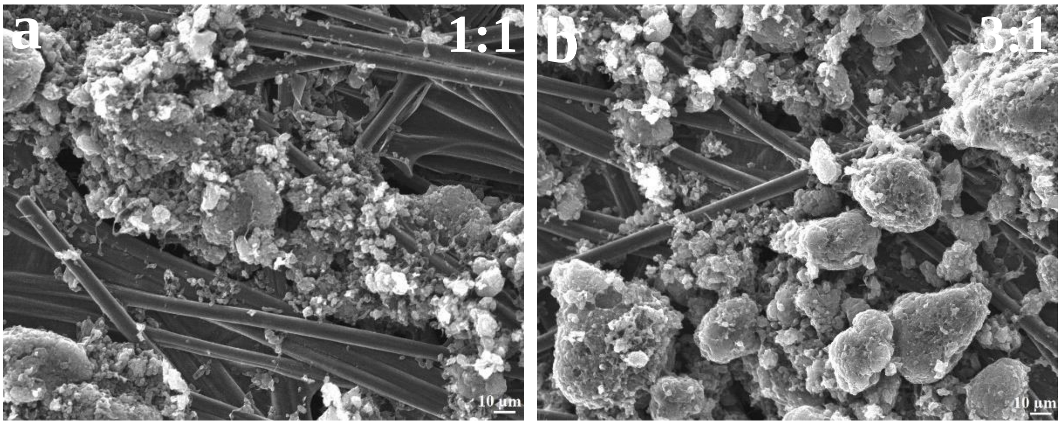

Figure S6. The surface morphology of the electrodes obtained treated by electrochemical oxidation without ultrasonication. Scanning electron microscopy (SEM) images of the electrodes fabricated at different PVF/CNT value. (a) 1:1, and (b) 3:1.

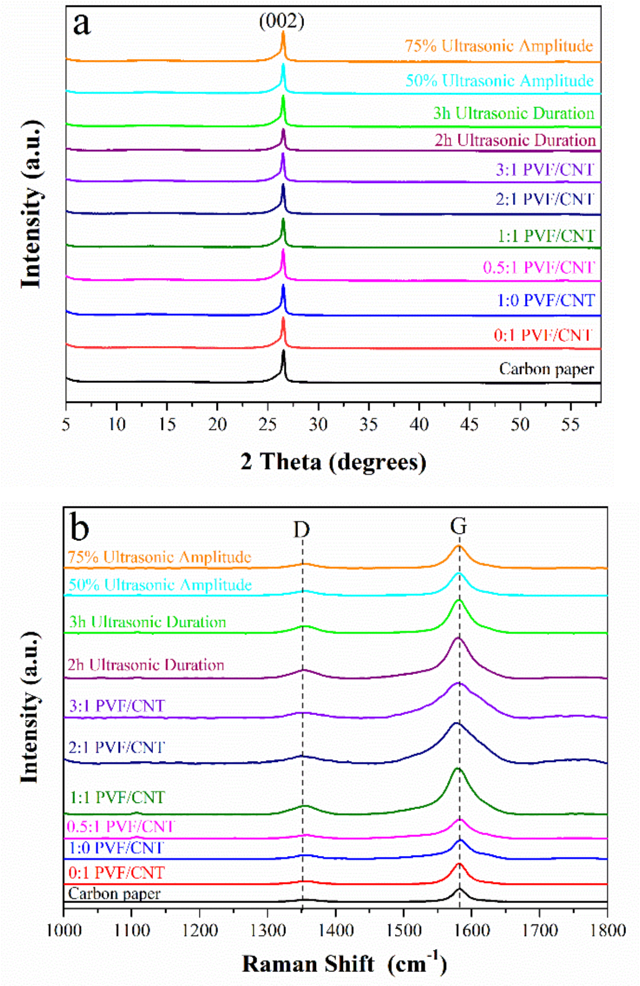

Figure S7. (a) X-ray diffraction (XRD) results, and (b) Raman results of the PVF/CNT electrodes prepared at different PVF/CNT, ultrasonic amplitude, and sonication duration conditions.
